# Association Between Multiple Chronic Conditions and Depressive Symptoms Among Older Adults in China: Evidence From the China Health and Retirement Longitudinal Study (CHARLS)

**DOI:** 10.3389/ijph.2023.1605572

**Published:** 2023-03-03

**Authors:** Haixia Liu, Zhongliang Zhou, Xiaojing Fan, Chi Shen, Yang Ma, Hongwei Sun, Zhaoyang Xu

**Affiliations:** ^1^ School of Public Health and Management, Binzhou Medical University, Yantai, Shandong, China; ^2^ School of Public Policy and Administration, Xi’an Jiaotong University, Xi’an, Shaanxi, China

**Keywords:** older adults in China, depressive symptoms, multiple chronic conditions (MCCs), comorbidity, urban and rural areas

## Abstract

**Objectives:** The purpose of this study was to explore the association between multiple chronic conditions (MCCs) and depressive symptoms among older adults in China.

**Methods:** We used the fourth round of data from the China Health and Retirement Longitudinal Study, and included 9789 older adults aged 60 years old and above.

**Results:** Compared with the older adults without chronic disease, older adults with MCCs and those with one chronic disease had a high risk of depression (MCCs: OR_-adjusted_: 1.55, 95% CI: 1.37 to 1.75; one chronic disease: OR_-adjusted_: 1.20, 95% CI: 1.05–1.37). In urban areas, the prevalence among older adults with MCCs was 2.01 times higher than that among older adults without chronic disease (OR_-adjusted_: 2.01, 95% CI: 1.56–2.60), while it was 1.44 times higher in rural areas (OR_-adjusted_:1.44, 95% CI: 1.25–1.65). Self-reported health, duration of sleep, social activities, and type of medical insurance were the influencing factors of depressive symptoms in older adults.

**Conclusion:** This study contributed to enriching the research on the relationship between MCCs and depressive symptoms in older adults in China.

## Introduction

Depression is a mental health problem characterized by persistent sadness, a loss of interest in some enjoyable activities, disturbed sleep and appetite, poor concentration, and other symptoms (1, 2). Studies have shown that when people transition from middle adulthood to older age, depressive symptoms tend to increase, often together with worsening physical health, which will cause a large disease burden with the increasing growth of the global aging population. Depression has been increasingly recognized as a serious public health concern in older populations (2–5). The Global Health Estimates Report showed that the prevalence of depression among elderly people had peaked, and more than 10% of elderly people worldwide have experienced depression (6). In China, the prevalence of depression in elderly people is approximately 15%, making depression the fourth leading cause of disability in China (7, 8).

The risk of depression, a psychiatric disorder that is related to physical health, is significantly related to the number of chronic diseases of an individual, thus the risk of depression increases with the number of chronic diseases. Hua’s study found that older adults with multiple chronic diseases (MCCs) often have different degrees of depressive symptoms (9–12). Presently, MCCs and health loss due to MCCs among older adults in China are gradually increasing (13, 14). Individual with MCCs usually require a combination of linked medical institutions for disease management from the initial screening until death, and they may need to take multiple drugs for a long time to control their conditions, which could lead to a decline in quality of life and could have varying influences on the risk of depression in older adults (11, 12). Meanwhile, Lichtman’s study showed that depression affected the occurrence, development, and prognosis of cardiovascular diseases, and it was listed as a risk factor for poor prognosis of patients with acute coronary syndrome (15), while hypertension, coronary heart disease, diabetes, and other chronic diseases were shown to be accompanied by a high incidence of depression among elderly individuals (16, 17). Thus, the risk of depression in older adults not only increases with the prevalence of physical diseases but is also related to severe neck, chest, and abdominal pain and many other chronic diseases. Mieke et al. found that the prevalence of depression in patients with chronic disease is much higher than that in the general population (11). Patients with MCCs were shown to have a higher degree of depression than those without MCCs.

In addition, due to China’s urban-rural dual economic structure, economic and social environments are different between urban and rural areas, which may have varying influences on the physical or mental health level of older adults. It is well known that there is a link between socioeconomic status and the development of disease through various mechanisms (18, 19). Urbanization, as an important manifestation of current social and economic factors, and the imbalance in social and economic development between urban and rural areas affect the differences in people’s habits and lifestyles, which leads to the heterogeneity of depression symptoms in older adults. Hu’s study showed that the age trajectories of later-life depression caseness varied by urbanization level. For both men and women and across all ages, the crude predicted probability of depression caseness was the highest in the rural group, followed by the semiurban group, and it was the lowest in the urban group (20). A meta-analysis based on 32 studies showed that the prevalence of depressive symptoms was approximately 10% higher in rural older adults aged 60 and above (29.2%) than that in their urban counterparts (20.5%) (21). Due to significant differences in the social and psychological status of elderly individuals in urban and rural areas, the proportion of elderly individuals who were at risk of developing depression was higher in rural areas (32.6%) than in urban areas (30.4%) (22). Jokela and Li’s studies found that the depression prevalence among middle-aged and older adults is lower with a higher level of urbanization (23), which was consistent with the result of Yang’s study that the prevalence rate of depression among the urban elderly individuals was lower than that of rural elderly individuals (31.9%) (24).

Dahlgren’s model of social determinants of health also pointed out that age, sex, lifestyle, social interaction, social experience and national policy environment were influencing factors of individual health (25). Wu’s study indicated that sex, age, marital status, social activities, and sleeping time and quality had significant impacts on depression in elderly individuals (26). Additionally, differences in intergenerational support received from children between urban and rural older adults receiving different intergenerational supports will have different impacts on depression in older adults, especially for those who are floating outside from their hometown (27). Jiang’s study found that self-reported health, social activities, education level and life satisfaction were influencing factors of depression in the older adults. The prevalence of depression varies significantly among older adults by self-reported health and life satisfaction (28). Therefore, we pose the following questions: after controlling for the social and environmental factors, what is the relationship between MCCs and depressive symptoms in older adults in China? After controlling for the confounding factors of socioeconomic and individual characteristics, especially when considering the background of China’s urban-rural dual structure, is there any difference in depressive symptoms between older adults living in urban and rural settings? Are there any differences in the relationship between MCCs and depressive symptoms between older adults living in urban and rural settings? Based on these questions, we propose the following two hypotheses:

Hypothesis 1 MCCs are an important influencing factor of depressive symptoms in older adults in China. The proportion of having a high risk of depression in older adults with MCCs is higher than that in older adults without MCCs.

Hypothesis 2 The relationship between MCCs and depressive symptoms in older adults is different between those living in urban and rural areas.

## Methods

### Data and Sample

**FIGURE 1:**
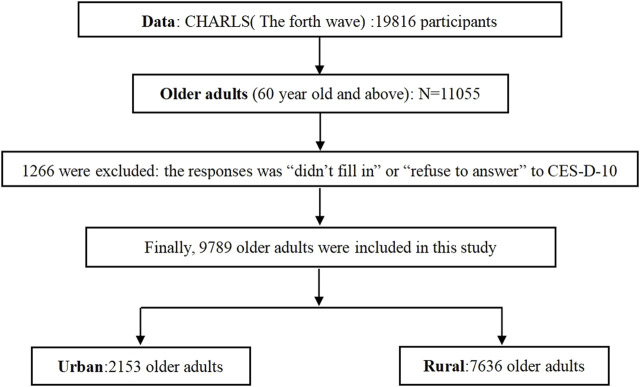
Study population screening process (China, 2018–2019).

Data were obtained from the fourth-round investigation of the China Health and Retirement Longitudinal Study (CHARLS), a large Chinese community study based on a sample of households with members aged 45 years or above. The investigation was conducted between July 2018 and March 2019 and involved 19817 respondents aged 45 years and above in 150 counties/districts and 450 villages/urban communities (3, 29, 30). According to the definition of older adults in China, “All citizens of the People’s Republic of China who have reached the age of 60 years belong to the older adults population,” 11055 older adults aged 60 years and above were selected as the research objects (the actual age of the older adults was calculated according to the question “What is your actual date of birth?”). According to the response of the core dependent variable (Depressive symptoms: The Center for Epidemiologic Studies Depression Scale, CES-D-10), if the respondents did not complete or refused to answer to the CES-D-10, they were removed from the study. Finally, 1266 older adults were excluded, and 9789 older adults were included in our study (for the sample screening process, see Figure 1).

## Measures

### Depressive Symptoms

Depressive symptoms of the participants were measured using the Center for Epidemiologic Studies Depression Scale (CES-D-10) (31, 32), which has been validated among the older adults and used in CHARLS (33). The CES-D-10 includes 10 questions regarding the participant’s experiences in the past week: feeling bothered, having trouble concentrating, feeling depressed, feeling as though everything required effort, feeling hopeful, feeling fearful, having restless sleep, feeling happy, feeling lonely, and having difficulty getting going (34). The total CES-D-10 score ranges from 0 to 30, with higher scores suggesting greater severity of depressive symptoms or a high risk of depression (35). In CHARLS, a cut-off score of 10 is usually used to distinguish participants (score≥ 10: high risk of depression, assigned to subgroup 1; score<10: low risk of depression, assigned to subgroup 0). In this study, the CES-D-10 had good reliability (Cronbach’s α = 0.799).

### Criteria of Chronic Disease and Multiple Chronic Conditions

In CHARLS, older adults with chronic disease are those who have been diagnosed with chronic disease by a doctor up to the time of the investigation, and the question is “Have you been diagnosed with [conditions listed below, read one by one] by a doctor?”. A total of 12 chronic diseases were listed and included in this study, including hypertension, dyslipidemia (high or low), diabetes or hyperglycemia, cancer, malignant tumor, chronic lung disease, liver disease, heart disease (heart infarction, coronary heart disease, angina pectoris, congestive heart failure, etc.), stroke (including cerebral infarction and cerebral hemorrhage), kidney disease (excluding tumor or cancer), diseases of the stomach or digestive system (excluding tumor or cancer), arthritis or rheumatism, and asthma. Based on the collection of chronic disease data, the criteria for MCCs in this study were defined as follows: if the older adults had two or more chronic diseases, they were defined as MCC patients. The variable of “MCCs or not” was defined as follows: if the older adults had two or more chronic diseases, they were assigned to subgroup 3; if they had one chronic disease, they were assigned to subgroup 2; and if they have no chronic disease, they were assigned to subgroup 1.

### Statistical Analysis

Descriptive analysis (N/%) was used to describe the status of MCCs and depressive symptoms of the older adults, and the chi-square test was used to compare the depressive symptoms between the older adults with MCCs and those without MCCs. Then, a generalized linear model (GLM) was employed to analyze the association between MCCs and depressive symptoms after controlling for other confounding factors, including analysis of urban and rural areas, and the results were expressed as adjusted odds ratios (OR_-adjusted_) and their 95% confidence intervals (95% CI).

GLM is an extension of the traditional linear model that allows the overall average to depend on a linear predictor through a non-linear link function and allows the response probability distribution to be any member of the exponential distribution family, including the logistic regression model. In this study, a binary logistic regression model was used to analyze the influence of MCCs on depressive symptoms in older adults in urban areas or rural areas after controlling for the confounding factors of sex, residence, education level, *per capita* household income, duration of sleep, social activities, self-reported health, type of medical insurance, life satisfaction, health satisfaction, marital satisfaction and child relationship satisfaction. A GLM always includes three components:

$$A\;linear\;component:\eta_{i}=\beta_{0}+\beta_{1}\chi_{1i}+\beta_{2}\chi_{1i}+\cdot \cdot \cdot +\beta_{m}\chi_{mi}$$ (1)

$$A\;random\;component:\varepsilon_{i}=Y_{i}-\eta_{i}$$ (2)

$$A\;link\;function:\eta_{i}=g\left(\mu_{i}\right)$$ (3)

In our study, we chose $g_{1}\left(\pi \right)=Ιn\left(\frac{\pi }{1-\pi }\right)$ as the link function; therefore, the form of the generalized linear model was:

$$\log it\left(\pi \right)=Ιn\left(\frac{\pi }{1-\pi }\right)=\beta_{0}+\beta_{1}X_{1}+\beta_{2}X_{2}+\cdot \cdot \cdot +\beta_{p}X_{p}$$ (4)

In Eq. 4, π is the prevalence rate of depressive symptoms in older adults, $\beta_{0}$ is a constant and $\beta_{p}$ is the regression coefficient, which represents the effect of $X_{p}$ on the predictive value of individual incidence π (Y = 1).

All statistical analyses were performed using IBM SPSS Statistics V22.0, and a result with a *p-value* < 0.05 was considered statistically significant.

## Results

The characteristics of the participants (9789 older adults) are displayed in Table 1. A total of 2,153 (22.0%) older adults lived in urban areas, and 7,636 (78.0%) lived in rural areas, including 4,933 (48.9%) male and 5,150 (51.1%) female older adults. A total of 30.7% of the older adults were aged 60–64 years old, 43.7% were aged 65–74 years old and 25.6% were aged over 75 years old. A total of 53.5% of the older adults had social activities, and 72.6% of the older adults stated that their self-reported health was “very good,” “good,” or “fair.” A total of 65.2% and 80.7% of the older adults were satisfied with their health and life, respectively. A total of 54.4% of the older adults suffered from MCCs, and 43.5% had a high risk of depression. There were significant differences in the prevalence of a high risk of depression between the older adults with or without MCCs, and the prevalence of a high risk of depression among the older adults with MCCs was higher than that of those with no chronic diseases or one chronic disease (χ^2^ = 399.59, *p* < 0.001). without controlling for other confounding factors, the prevalence of a high risk of depression among older adults with MCCs was 1.84 times higher than that of the older adults who had no chronic diseases without controlling for other factors. Meanwhile, the prevalence of a high risk of depression among older adults living in rural setting was higher than that among older adults living in an urban setting (χ^2^ = 5.88, *p* < 0.05). The prevalence of a high risk of depression among older adults with no social activities was higher than that among older adults who had social activities (χ^2^ = 40.41, *p* < 0.001). There were also significant differences in the prevalence of a high risk of depression among the older adults in different groups of self-reported health, duration of sleep, *per capita* household income, and medical insurance types (Table 2).

**TABLE 1:** The characteristics of the study participants (China, 2018–2019).

Variables	Options	N (%)	Variables	Options	N (%)
Residence	Urban	2,153 (22.0)	Duration of sleep	≤5 h	3,317 (33.9)
	Rural	7,636 (78.0)		6–9 h	6,055 (61.9)
Sex	Male	4,779 (48.8)		≥10 h	417 (4.3)
	Female	5,010 (51.2)	Social activities	Yes	5,237 (53.5)
Age(year)	60-64	3,009 (30.7)		No	4,552 (46.5)
	65-74	4,273 (43.7)	Per capita household income	Low income	3,728 (38.1)
	≥75	2,507 (25.6)		Middle income	4,858 (49.6)
Marital status	Married and living with spouse	7,287 (74.4)		High income	1,203 (12.3)
	Married, not living with spouse	365 (3.7)	Life satisfaction	Satisfied	7,900 (80.7)
				Dissatisfied	1889 (19.3)
	Divorced	94 (1.0)	Health satisfaction	Satisfied	6,387 (65.2)
	Widowed	1974 (20.2)		Dissatisfied	3,402 (34.8)
			Marital satisfaction	Satisfied	7,727 (78.9)
	Never married	69 (0.7)		Dissatisfied	846 (8.6)
Education level	Illiterate	3,103 (31.7)	Child relationship satisfaction	Satisfied	9247 (94.5)
	Primary school and below	4,256 (43.5)		Dissatisfied	453 (4.6)
	Elementary school and above	2,430 (24.8)	Type of medical insurance	Urban employees	1,320 (13.5)
Self-reported health	Very good	1,068 (10.9)		Urban and rural residents	7,504 (76.7)
	Good	1,225 (12.5)		Free	617 (6.3)
	Fair	4,812 (49.2)		Commercial insurance and others	348 (3.6)
	Poor	2016 (20.6)	Chronic disease	Yes	7,704 (78.7)
	Very poor	653 (6.7)		No	2085 (21.3)
Depression	High risk of depression	4,258 (43.5)	MCCs	No chronic disease	2088 (21.3)
	Low risk of depression	5,531 (56.5)		One chronic disease	2,376 (24.3)
				MCCs	5,325 (54.4)

**TABLE 2:** Comparison of the prevalence of depressive symptoms in older adults (China, 2018–2019).

Variables	Option	High risk of depression (N/%)	Low risk of depression (N/%)	*χ* ^*2*^_*-value*_
MCCs or not	No chronic disease	592 (28.4)	1,496 (71.6)	399.59**
	Having one chronic disease	884 (37.2)	1,492 (62.8)	
	Having MCCs	2,783 (52.3)	2,542 (47.7)	
Sex	Male	2058 (43.1)	2,721 (56.9)	0.75
	Female	2,201 (43.9)	2,809 (56.1)	
Residence	Urban	3,273 (42.9)	4,363 (57.1)	5.88*
	Rural	986 (45.8)	1,167 (54.2)	
Age (years)	60–64	1,295 (43.0)	1714 (57.0)	0.46
	65–74	1873 (43.8)	2,400 (56.2)	
	≥75	1,091 (43.5)	1,416 (56.50	
Marital status	Married and living with spouse	3,175 (43.6)	4,112 (56.4)	0.66
	Married, not living with spouse	152 (41.6)	213 (58.4)	
	Divorced	40 (42.6)	54 (57.4)	
	Widowed	863 (43.7)	1,111 (56.3)	
	Never married	29 (42.0)	40 (58.0)	
Education level	Illiterate	1,370 (44.2)	1739 (55.8)	2.68
	Primary school and below	1866 (43.8)	2,390 (56.2)	
	Elementary school and above	1,023 (42.1)	1,407 (57.9)	
Self-reported health	Very good	217 (20.3)	851 (79.7)	1,063.75**
	Good	312 (25.5)	913 (74.5)	
	Fair	1921 (39.9)	2,891 (60.1)	
	Poor	1,316 (65.3)	700 (34.7)	
	Very poor	486 (74.4)	167 (5.6)	
Per capita household income	Low income	1739 (46.6)	1989 (53.4)	28.51**
	Middle income	2052 (42.2)	2,806 (57.8)	
	High income	468 (38.9)	735 (61.1)	
Duration of sleep	≤5 h	1955 (58.9)	1,362 (41.1)	487.42**
	6–9 h	2,144 (35.4)	3,911 (64.5)	
	≥10 h	160 (38.4)	257 (61.6)	
Social activities	No	2,136 (46.9)	2,416 (53.1)	40.41**
	Yes	2,123 (40.5)	3,114 (59.5)	
Life satisfaction	Satisfied	828 (43.8)	1,061 (56.2)	0.10
	Dissatisfied	3,431 (43.4)	4,469 (56.6)	
Health satisfaction	Satisfied	1,458 (42.9)	1944 (57.1)	0.90
	Dissatisfied	2,801 (43.9)	3,586 (56.1)	
Marital satisfaction	Satisfied	360 (42.6)	486 (57.4)	0.43
	Dissatisfied	3,364 (43.5)	4,363 (56.5)	
	Never married	535 (44.0)	681 (56.0)	
Child relationship satisfaction	Satisfied	185 (40.8)	268 (59.2)	1.75
	Dissatisfied	4,038 (43.7)	5,209 (56.3)	
	No child	36 (40.4)	53 (59.6)	
Type of medical insurance	Urban employees	471 (35.7)	849 (64.3)	40.83**
	Urban and rural residents	3,379(45.0)	4,125 (55.0)	
	Free	257 (41.7)	360 (58.3)	
	Commercial insurance and others	152 (43.7)	196 (56.3)	

Note: *p < 0.05; **p < 0.01.

After controlling for the variables of sex, residence, age, marital status, education level, self-reported health, *per capita* household income, duration of sleep, life satisfaction, health satisfaction, child relationship satisfaction, and type of medical insurance (Table 3), the prevalence of a high risk of depression among the older adults with MCCs was 1.55 times higher than that in the older adults without chronic disease (OR_-adjusted_: 1.55, 95% CI: 1.37–1.75), and the prevalence of high risk of depression among the older adults with one chronic disease was 1.20 times higher than that of the older adults without chronic disease (OR_-adjusted_: 1.20, 95% CI: 1.05–1.37), which supported the first hypothesis of our study (MCCs are an important influencing factor of depressive symptoms in older adults in China. The proportion of having high a risk of depression in older adults with MCCs is higher than that in older adults without MCCs).

**TABLE 3:** Influence of having multiple chronic conditions on the risk of depression among older adults (China, 2018–2019).

Variables	Options	Model I		Model II	
		OR	OR (95% CI)	OR	OR (95% CI)
MCCs or not	No chronic disease	-	-	1	-
	Having one chronic disease	-	-	1.20**	1.05, 1.37
	Having MCCs	-	-	1.55**	1.37, 1.75
Sex	Male	1	-	1	-
	Female	1.01	0.92, 1.12	1.02	0.93, 1.23
Residence	Urban	1	-	1	-
	Rural	1.12*	1.01, 1.24	1.13*	1.01, 1.25
Age (years)	60–64	1	-	1	-
	65–74	1.05	0.94, 1.16	1.05	0.94, 1.16
	≥75	1.01	0.89, 1.14	1.01	0.89, 1.14
Marital status	Married and living with spouse	1	-	1	-
	Married, not living with spouse	1.01	0.80, 1.27	1.01	0.80, 1.28
	Divorced	0.98	0.62, 1.55	1.01	0.64, 1.60
	Widowed	1.01	0.87, 1.16	1.00	0.87, 1.16
	Never married	0.90	0.52, 1.55	0.90	0.52, 1.55
Education level	Illiterate	1	-	1	-
	Primary school and below	0.98	0.88, 1.10	0.98	0.88, 1.09
	Elementary school and above	0.91	0.80, 1.03	0.91	0.80, 1.03
Self-reported health	Very good	1	-	1	-
	Good	1.30**	1.07, 1.59	1.25*	1.03, 1.53
	Fair	2.40**	2.04, 2.82	2.15**	1.82, 2.53
	Poor	6.23**	5.21, 7.44	5.13**	4.26, 6.17
	Very poor	9.57**	7.57, 12.10	7.68**	6.03, 9.77
Per capita household income	Low income	1	-	1	-
	Middle income	0.92	0.84, 1.01	0.92	0.84, 1.01
	High income	0.91	0.78, 1.07	0.91	0.78, 1.07
Duration of sleep	≤5 h	1	-	1	-
	6–9 h	0.45**	0.41, 0.49	0.46**	0.42, 0.51
	≥10 h	0.48**	0.39, 0.60	0.49**	0.39, 0.62
Social activities	No	1	-	1	-
	Yes	0.88**	0.81, 0.96	0.87**	0.80, 0.95
Life satisfaction	Dissatisfied	1	-	1	-
	Satisfied	0.95	0.84, 1.09	0.95	0.84,1.09
Health satisfaction	Dissatisfied	1	-	1	-
	Satisfied	1.03	0.92, 1.15	1.03	0.92, 1.15
Marital satisfaction	Dissatisfied	1	-	1	-
	Satisfied	1.04	0.92, 1.18	1.05	0.93, 1.18
Child relationship satisfaction	Dissatisfied	1	-	1	-
	Satisfied	1.12	0.91, 1.36	1.12	0.92, 1.37
Type of medical insurance	Urban employees	1	-	1	-
	Urban and rural residents	1.28**	1.11, 1.47	1.29**	1.12, 1.48
0	Free	1.23	1.00, 1.52	1.23	0.99, 1.52
	Commercial insurance and others	1.28	0.98, 1.67	1.29	0.99, 1.69

Taking the first category as the reference category (the one with the minimum value).

OR: *OR*_-adjusted_ odds ratio with other variables controlled; 95% CI: 95% confidence interval.

*p < 0.05, **p < 0.01.

Showcasing the impact of urban-rural difference on depression in older adults, after controlling for MCCs and other confounding factors the prevalence of a high risk of depression among older adults living in rural areas was 1.13 times higher than that among older adults living in urban areas (OR_-adjusted_: 1.13, 95% CI: 1.01–1.25), which indicated that there were differences in depressive symptoms between older adults in urban and rural settings. Meanwhile, whether in urban or rural areas, having MCCs was an influencing factor of depressive symptoms in older adults (Table 4). The prevalence of a high risk of depression in the older adults with MCCs was 2.01 times higher than that among older adults without chronic disease and living in urban areas (OR_-adjusted_ = 2.01, 95% CI: 1.56–2.60), while it was 1.44 times higher than that of older adults living in rural areas (OR_-adjusted_ = 1.44, 95% CI: 1.25–1.65), which supported hypothesis 2 of our study (The relationship between MCCs and depressive symptoms in older adults is different between those living in urban and rural areas). In rural areas, the prevalence of a high risk of depression in the older adults with one chronic disease was 1.18 times higher than that in older adults without chronic disease and living in urban areas (OR_-adjusted_ = 1.18, 95% CI: 1.01–1.37). The variables of self-reported health, sleep duration, social activities, and type of medical insurance were influencing factors of depressive symptoms among older adults in both urban and rural areas.

**TABLE 4:** Influence of having multiple chronic conditions on the risk of depression among older adults in urban and rural areas (China, 2018–2019).

Variables	Options	Urban		Rural	
		OR	OR (95% CI)	OR	OR (95% CI)
MCCs or not	No chronic disease	1	-	1	-
	Having one chronic disease	1.27	0.95, 1.68	1.18*	1.01, 1.37
	Having MCCs	2.01**	1.56, 2.60	1.44**	1.25, 1.65
Sex	Male	1	-	1	-
	Female	0.94	0.76, 1.16	1.04	0.94, 1.16
Age	60–64	1	-	1	-
	65–74	1.02	0.82, 1.28	1.06	0.94, 1.19
	≥75	0.85	0.65, 1.11	1.06	0.92, 1.22
Marital status	Married and living with spouse	1	-	1	-
	Married, not living with spouse	1.04	0.65, 1.67	1.02	0.78, 1.33
	Divorced	1.61	0.60, 4.33	0.88	0.52, 1.49
	Widowed	1.09	0.80, 1.47	0.99	0.84, 1.17
	Never married	0.89	0.29, 2.74	0.93	0.50, 1.75
Education level	Illiterate	1	-	1	-
	Primary school and below	1.01	0.80, 1.28	0.97	0.86, 1.10
	Elementary school and above	0.82	0.62, 1.09	0.93	0.80, 1.07
Self-reported health	Very good	1	-	1	-
	Good	1.21	0.79, 1.86	1.28*	1.02, 1.61
	Fair	2.23**	1.56, 3.16	2.12**	1.76, 2.56
	Poor	4.89**	3.30, 7.26	5.23**	4.24, 6.45
	Very poor	4.75**	2.86, 7.88	8.82**	6.70, 11.62
Per capita household income	Low income	1	-	1	-
	Middle income	0.98	0.80, 1.20	0.91	0.81, 1.01
	High income	1.18	0.84, 1.66	0.85	0.71, 1.01
Duration of sleep	≤5 h	1	-	1	-
	6–9 h	0.46**	0.37, 0.56	0.46**	0.42, 0.52
	≥10 h	0.43**	0.27, 0.67	0.51**	0.40, 0.66
Social activities	No	1	-	1	-
	Yes	0.80*	0.67, 0.97	0.90*	0.82, 0.99
Life satisfaction	Dissatisfied	1	-	1	-
	Satisfied	0.99	0.75, 1.32	0.94	0.81, 1.10
Health satisfaction	Dissatisfied	1	-	1	-
	Satisfied	1.13	0.89, 1.42	1.00	0.89, 1.13
Marital satisfaction	Dissatisfied	1	-	1	-
	Satisfied	0.91	0.71, 1.18	1.09	0.94, 1.25
Child relationship satisfaction	Dissatisfied	1	-	1	-
	Satisfied	1.02	0.66, 1.58	1.13	0.90, 1.42
Type of medical insurance	Urban employees	1	-	1	-
	Urban and rural residents	1.61**	1.14, 2.29	1.22*	1.04, 1.43
	Free	1.55	0.94, 2.55	1.28	0.93, 1.49
	Commercial insurance and others	1.42	0.86, 2.34	1.30	0.93, 1.80

Taking the first category as the reference category (the one with the minimum value).

OR: *OR*_-adjusted,_ odds ratio with other variables controlled; 95% CI: 95% confidence interval.

*p < 0.05, **p < 0.01.

## Discussion

These analyses added important information to understand the relationship between MCCs and depression among the older adults in China, using a nationally representative cross-sectional dataset of CHARLS. We found that there was a significant difference in the prevalence of a high risk of depression between older adults with MCCs and those without chronic disease, and the prevalence of a high risk of depression in older adults with MCCs was significantly higher than that in older adults without chronic disease. The results indicated that MCCs were an important influencing factor on depressive symptoms in older adults, which was consistent with the results of Meher’s study, which showed that the association between the number of chronic conditions and depression exhibited a linear trend with an increased odds ratio indicating a higher risk of depression among older adults with multiple chronic conditions (36). Our study found that 52.3% of the older adults with MCCs had a high risk of depression, and the prevalence of a high risk of depression among the older adults without chronic disease was 27.4%. Other studies also indicated that older adults with chronic diseases often have different degrees of depression, and the number of chronic diseases was positively related to the score of depressive symptoms: the more MCCs, the higher the score of depressive symptoms (37–40). Usually, MCCs among older adults have a long course, are difficult to cure, and easily recur. Some patients need long-term or even lifelong treatment, which aggravates the patient’s economic pressure, thus increasing the mental pressure and psychological disorder of older patients. The risk of depression in older adults with cerebrovascular disease, coronary heart disease, diabetes, and visual impairment is higher than that in older adults without those chronic diseases (39, 40). Depressive symptoms were associated with increased medical utilization and expenditure among chronic lung disease patients, which varies between urban and rural areas (41).

In particular, under the background of rapid urbanization, the proportion of the urban population increased from 26% in 1990% to 60.6% in 2019 in China. Moving to urban areas is viewed as a means of upwards social mobility in China, as it will lead to a better educational environment, more economic resources, more opportunities, and a sense of cultural and psychological preeminence (41). The difference between residing in urban and rural areas has different effects on depression symptoms in older adults. Our study showed that urban versus rural residences were the influencing factors of depression symptoms in older adults after controlling for other confounding factors. The prevalence of a high risk of depression among older adults living in rural areas was 1.13 times higher than that among older adults living in urban areas, which was consistent with the results of some previous studies (41–43).

Whether in urban or rural areas, the variables of self-reported health, duration of sleep, social activities, and type of medical insurance were the influencing factors of depressive symptoms in older adults; the worse their self-reported health, the higher the risk of depression in older adults, which was consistent with the results of Maier et al.’s study. In their meta-analysis, the results of the heterogeneity analysis indicated that self-reported health had a significant influence in several studies. Quality of physical activity, chronic disease, and difficulty initiating sleep increased the risk of depression (44). The risk of depression among older adults was also associated with the duration of sleep. Our study also found that the prevalence of a high risk of depression among older adults who slept 6–9 h every day was 0.46 times higher than that among the older adults who slept for less than 6 h, and the prevalence of a high risk of depression among older adults who slept 10 or more hours every day was 0.49 times higher than that among older adults who slept for less than 6 h. The prevalence of high a risk of depression among the older adults who slept less than or equal to 5 h was the highest, followed by those who slept more than or equal to 10 h, and the prevalence of high risk of depression among the older adults who slept for 6–9 h was the lowest. The results indicated that proper sleep time could reduce the risk of depression in elderly individuals, which was similar to the results of Zeo’s study to some extent. Compared with older adults who had less than 5 h sleep, those who had more sleep had a lower risk of depression (28, 45). The duration of sleep was a significant influencing factor against depressive symptoms in old people. Their study also pointed out that the quality of sleep affected the risk of depressive symptoms and moderated the relationship between pain interference and depressive symptoms among older adults with chronic pain, such that good sleep quality attenuated the effect of pain interference on depression, and poor sleep quality amplified the effect of pain interference on depression (45–47).

Meanwhile, we found that social activities could reduce the risk of depression in older adults. The prevalence of a high risk of depression among people who participated in social activities was lower than that among older adults who did not participate in social activities, whether in rural or urban areas. The results in Wang’s study indicated that for urban respondents, playing mah-jong or cards and joining sports or social clubs predicted a decline in depressive symptoms. For rural residents, interacting with friends regularly was associated with fewer depressive symptoms. In conclusion, a more diverse and higher frequency of social participation was associated with better mental health, while the social significance of social participation varied across different types of social participation and between rural and urban areas (48). Therefore, social activities play an important role in depressive symptoms among older adults. We can try to encourage and provide social opportunities for older adults based on their personal needs, preferences and abilities, actively build entertainment and leisure service facilities, and encourage older adults to participate in more social activities, which may reduce the risk of depression to some extent.

### Conclusion

Our study found that whether they were living in urban or rural areas, MCCs were important factors influencing depressive symptoms among older adults. Compared with older adults without MCCs, older adults with MCCs have a higher risk of depression, and the influence of MCCs on depressive symptoms in older adults is different between urban and rural areas. This study added important information to help us understand the relationship between MCCs and depressive symptoms in older adults in China. However, there are also some limitations: (1) We analyzed the relationship between MCCs and depressive symptoms among older adults based on a cross-sectional study, not on a cohort study. Therefore, the evidence of this study was weak regarding the determination of a causal relationship. (2) This study only focused on the impact of MCCs on depressive symptoms in older adults, but did not consider the impact of different types of chronic disease and different combinations of chronic diseases. The impact of different types and combinations of chronic diseases on depressive symptoms needs in-depth study in the future. (3) Although the CES-D-10 has been widely used in the investigation of depressive symptoms and has good reliability, it was based on the subjective feelings of participants and not a diagnosis from a doctor. Therefore, the results from the screening tool may include some false-positives.
